# An Insecure Role for Securin in Chromosome Segregation

**DOI:** 10.1371/journal.pbio.0030437

**Published:** 2005-11-29

Just as good parents try to prevent inequities among their progeny, a dividing cell must ensure that its daughters inherit all the chromosomes they are entitled to. But with cells, such evenhandedness goes beyond matters of equity to those of life and death. The progeny of cancer cells, for example, typically suffer rampant chromosome losses. Though these losses should eventually cause the cells' demise, they can also reveal or induce mutations that encourage proliferation, which explains why cancer cells accommodate widespread chromosomal instability. Understanding how normal cells unerringly transmit a full chromosome set to their daughters has long been an important part of the fight against cancer.

In the ballet of cell division, or mitosis, chromosomes, intracellular fibers, and the cell outer membrane execute carefully choreographed steps and partner shifts. First, cells replicate their DNA, creating twin sets of each chromosome, known as sister chromatids. Mitosis, per se, starts when the chromatids condense their DNA into compact bodies that are captured in a spindle of tubulin fibers (called microtubules). The microtubules first line up the chromatid pairs along the spindle's middle plane; then they pull them apart, hauling the members of each pair to opposite ends of the spindle. The mother cell then pinches its membrane along the spindle's middle plane, splitting into two daughter cells with full chromosome sets. Keeping the chromatids of a pair together at the beginning of mitosis, and allowing their timely separation at the end, are both crucial steps for proper chromosome segregation into the daughter cells.

From the time DNA replication begins, sister chromatids are held together by a protein complex named cohesin. After the chromatid pairs are all neatly positioned in the center of the spindle, they can be safely segregated, a job performed by separase, an enzyme that dissolves cohesin's grip by breaking down one of its protein components. The dissolving power of separase is tightly controlled to avoid precocious or delayed chromatid separation. One of separase's regulators is securin, a protein known as a chaperone that appears to hold separase captive until just the right time. A recent study indicated that human cells devoid of securin underwent abnormal mitoses that led to widespread chromosome losses, making securin a key player in chromosome segregation and a promising entry point into cancer therapy. But in a new study in *PLoS Biology*, Katrin Pfleghaar, Michael Speicher, and colleagues have repeated and expanded on these experiments, and come to somewhat different conclusions.

Both teams carried out the same experiments in the same system: they counted chromosomes and examined the mitotic process in cultures of human cells lacking the securin gene. And both teams found that in the first weeks of cell culture, cells were losing chromosomes at a very high rate and most mitoses showed abnormal chromatid distribution. But in this study, Pfleghaar observed the cells for longer periods and found that as time went by, the culture recovered: cells with abnormal mitoses and chromosome counts became rarer until, after a few weeks, the cells appeared indistinguishable from their relatives with an intact securin gene. Interestingly, both studies found that the amounts and activity levels of separase were low in securin-deficient cells, which confirms that securin regulates separase. Pfleghaar and her colleagues speculate that securin normally plays an important role in mitoses, but that in its absence, cells tap into compensatory mechanisms to restore proper chromosome segregation.

The implications for cancer treatment are potentially great, as mathematical models of cancer growth do not usually include the possibility that cell populations might recover from chromosomal instability. In addition, such recoveries might interfere with therapies that aim to kill cancer cells by exacerbating their chromosome losses. —*Francoise Chanut*

